# Population attributable fraction of Esophageal squamous cell carcinoma due to smoking and alcohol in Uganda

**DOI:** 10.1186/s12885-016-2492-x

**Published:** 2016-07-11

**Authors:** Samson Okello, Cristina Churchill, Rogers Owori, Benson Nasasira, Christine Tumuhimbise, Charles Lagoro Abonga, David Mutiibwa, David C. Christiani, Kathleen E. Corey

**Affiliations:** Department of Internal Medicine, Mbarara University of Science and Technology, P. O Box 1410 Mbarara, Uganda; Department of Epidemiology, Harvard T.H Chan School of Public Health, Boston, MA USA; Department of Medicine, University of Virginia, Charlottesville, VA USA; Gwatiiro Hospital, Wakiso, Uganda; Department of Surgery, Mbarara University of Science and Technology, Mbarara, Uganda; Department of Environmental Health, Harvard T.H Chan School of Public Health, Boston, MA USA; Harvard Medical School, Massachusetts General Hospital, Boston, USA; Department of Medicine, Massachusetts General Hospital, Boston, MA USA

**Keywords:** Population attributable fraction, Esophageal squamous cell carcinoma, Alcohol, Smoking, Africa

## Abstract

**Background:**

Despite the high rates and regional variation of esophageal squamous cell carcinoma (ESCC) in East Africa, the contributions of smoking and alcohol to the ESCC burden in the general population are unknown.

**Methods:**

We conducted a case-control study of patients presenting for upper gastrointestinal endoscopic examination at Mbarara Regional Referral Hospital, Uganda. Sociodemographic data including smoking and alcohol intake were collected prior to endoscopy. Cases were those with histological diagnosis of ESCC and controls were participants with normal endoscopic examination and gastritis/duodentitis or normal histology. We used odds ratios associated with ESCC risk to determine the population attributable fractions for smoking, alcohol use, and a combination of smoking and alcohol use among adults aged 30 years or greater who underwent upper gastrointestinal endoscopy.

**Results:**

Our study consisted of 67 cases and 142 controls. Median age was 51 years (IQR 40–64); and participants were predominantly male (59 %). Dysphagia and/or odynophagia as indications for endoscopy were significantly more in cases compared to controls (72 % vs 6 %, *p* < 0.0001). Male gender and increasing age were statistically associated with ESCC. In the unadjusted models, the population attributable fraction of ESCC due to male gender was 55 %, female gender - 49 %, smoking 20 %, alcohol 9 % and a combination of alcohol & smoking 15 %. After adjusting for gender and age, the population attributable fraction of ESCC due to smoking, alcohol intake and a combination of alcohol & smoking were 16, 10, and 13 % respectively.

**Conclusion:**

In this population, 13 % of esophageal squamous cell carcinoma cases would be avoided if smoking and alcohol use were discontinued. These results suggest that other important risk factors for ESCC in southwestern Uganda remain unknown.

## Background

Esophageal squamous cell carcinoma is lethal, with a poor 5-year survival even in resource rich settings [1, 2]. In sub-Saharan Africa, the incidence of esophageal squamous cell carcinoma (ESCC) has regional variation with southern and eastern regions reporting significantly higher incidence rates [3]. It is postulated that differences in risk factor profiles may explain these regional variations. Therefore, public health approaches for risk factor modification and early screening methods for curable lesions offer a great opportunity to reduce ESCC morbidity and mortality.

Though alcohol use and smoking are known risk factors for ESCC [4], existing data are inconclusive in linking various factors to an increased risk of ESCC including: human papilloma virus [5, 6], consumption of hot drinks [7], diets low in fruit and vegetables [8], and exposure to nitrosamines [9, 10], suggesting that these environmental risk factors alone may not be responsible for ESCC.

Though alcohol and tobacco are thought to synergistically interact for the development of esophageal squamous cell carcinoma [11], data are lacking in the characterizations of the impact of these risk factors on the incidence of ESCC using population attributable fractions (PAFs). PAFs account both for the strength of the association and the prevalence of the risk factor. Studies in ESCC among low and high risk populations report between 60 and 75 % population attributable fractions for ESCC due to smoking and alcohol [12–15] but PAFs for Africa have not been previously reported. We aimed to determine the population attributable fraction for smoking and alcohol use for ESCC risk in an ESCC high incidence African population in southwestern Uganda.

## Methods

### Study participants and procedures

We carried out a case control study using data from all patients, aged 30 years or greater, who presented for upper gastrointestinal endoscopy from January 2003 to December 2014 at the Mbarara Regional Referral Hospital (MRRH), southwestern Uganda. Our endoscopy service was nonfunctional during 2010 to 2012. However, our center is the only endoscopy service in southwestern Uganda; receiving referrals from within MRRH and other health facilities; private and public in southwestern Uganda with an estimated population of 8.7 million people [16].

Before performing upper GI endoscopy, a trained nurse administered a standardized questionnaire to collect data including age, gender, history of alcohol and smoking, and symptoms after provision of informed consent for endoscopy & biopsy.

A nurse sprayed 5 mL of 1 % lidocaine into the patients’ mouth for 5 min; intravenous medications diazepam 5 mg and fentanyl 50 μg were then administered for sedation. With the patients positioned in the left lateral position, a trained endoscopist then inserted a gastroscope visually examining the entire esophagus and stomach. Tissue biopsies of both abnormal and occasionally normal mucosae were picked from unstained foci with the number of biopsies taken depended on the size of the lesion but minimum of three biopsies. Some pieces of tissue biopsy were used to perform rapid urease (CLO) test while the remaining biopsies were kept in 10 % buffered formalin before transportation to the histopathology laboratory at the department of Pathology, Mbarara University of Science and Technology (MUST) for histopathology microscopic examination.

At the MUST histopathology laboratory, all esophageal biopsies were processed into sections, stained with hematoxylin and eosin for 1 h, and then examined using standard diagnostic criteria for microscopic atypia. Specifically, for esophageal squamous cell carcinoma, pathologist reported features such as presence of nuclear atypia, prominent keratinization and evidence of invasion [17].

### Data collection

We reviewed participants’ records, first extracting cases of esophageal squamous cell carcinoma and then consecutively sampling for controls. For all participants, we collected pre-endoscopic data including age, sex, history of alcohol and smoking, and presenting symptoms. We also reviewed diagnostic upper gastrointestinal endoscopy reports to collect information pertaining to the site of suspicious esophageal lesions i.e., upper third (15–24 cm), middle third (24–32 cm), and lower third (32–40 cm) esophageal lesions [18]. All data were doubly entered into an electronic database and crosschecked to minimize entry and transcription errors.

### Ascertainment of cases and controls

We defined a case as any participant having histological diagnosis of esophageal squamous cell carcinoma either differentiated or undifferentiated. Controls were participants with normal endoscopic examination or gastritis/duodentitis or normal histology. We excluded from this analysis participants with histological diagnoses of other esophageal abnormalities such as esophageal dysplasia, esophageal hyperplasia, esophagitis, esophageal adenocarcinoma, Barrett’s esophagus or gastric cancer, peptic ulcer disease or missing histology results.

### Statistical analysis

We described baseline characteristics of cases and controls using summary statistics.

Associations between exposure variables: sex, age at presentation, history of smoking and alcohol with esophageal squamous cell carcinoma were estimated in unadjusted and multivariate logistic regression models expressed as odds ratios with 95 % confidence intervals. Exposure variables smoking and drinking alcohol were treated as binary (never, reference group; ever, risk group). Only variables that provided odds ratios (ORs) for ESCC with *p*-values < 0.1 in the univariate logistic regression model were included in the multivariate logistic regression model.

Because the main objective of this analysis was to investigate the population attributable fraction of Esophageal squamous cell carcinoma due to smoking and alcohol, we assumed that smoking and drinking alcohol individually exert effects on ESCC risk and that these interact thus, we introduced a joint category of smoking and alcohol using an interaction term [19]. We used crude odds ratio estimates from the univariate logistic regression and the adjusted odds ratios controlling for gender and age to determine the unadjusted and adjusted population attributable fractions (PAF) for smoking, alcohol use, and a combination of smoking and alcohol use. We used crude odds ratio estimates from the univariate logistic regression and the adjusted odds ratios controlling for gender and age to determine the unadjusted and adjusted population attributable fractions (PAF) for smoking, alcohol use, and a combination of smoking and alcohol use respectively. This was calculated using the punafcc package available in STATA software. The PAFs and 95 % confidence intervals were estimated by a method that is based on unconditional logistic regression [20], which provides adjusted PAF estimates by combining adjusted odds ratio estimates and the observed prevalence of the risk factors among case patients.

All statistical tests were two-sided. All analyses were performed with STATA version 13 (Stata Corporation, College Station, Texas, USA).

## Results

Among 913 endoscopes performed at Mbarara Regional Referral Hospital between 2003 and 2014, there were 67 (7.34 %) new cases of esophageal squamous cell carcinoma. We excluded 428 (46.88 %) with missing histology results, 53 (5.81 %) with inadequate biopsies (no histological diagnosis could be made), and 148 with other histology results of esophagitis, esophageal adenocarcinoma, Barrett’s esophagus, esophageal Kaposi sarcoma, and gastric malignancies.

Among the 142 controls, 30 (21.13 %) had normal histology and 45 (31.69 %) had gastritis/duodentitis. There were a higher proportion of males among cases compared to controls (79.10 % vs 52.82 %). The age distribution was similar among cases and controls except for < 41 years with a higher proportion of controls (Table 1).

**Table 1:** Baseline characteristics of cases and controls

Characteristic	Control	Case
	*n* = 142, *n* (%)	*n* = 67, *n* (%)
Male gender	75 (52.82)	53 (79.10)
Age group, years		
31–40	29 (20.42)	2 (2.99)
41–50	28 (19.72)	19 (28.36)
51–60	32 (22.54)	16 (23.88)
61–70	27 (19.01)	16 (23.88)
>70	22 (15.49)	11 (16.42)
Missing	4 (2.82)	3 (4.48)
Substance abuse		
Smoking	8 (5.63)	6 (8.96)
Alcohol	22 (15.49)	6 (8.96)
Alcohol & smoking	10 (7.04)	14 (20.90)
Presenting symptoms		
Dyspepsia	77 (54.23)	_
Dysphagia	14 (9.86)	38 (56.72)
Dysphagia & weightloss	4 (2.82)	6 (8.96)
Regurgitation^b^	8 (5.63)	1 (1.49)
Odynophagia	4 (2.82)	8 (11.94)
Upper GI bleeding^a^	16 (11.27)	_
Others^c^	18 (12.68)	6 (8.96)
Dysphagia & Odynophagia	1 (0.70)	8 (11.94)
HIV positive	2 (1.41)	2 (2.99)

Upper GI bleeding^a^:hematemesis and/or melena; Regurgitation^b^: Epigastric pain, heartburn, and/or regurgitation Others^c^:vomiting, abdominal pain, and chest pain

As expected, indication for endoscopy were significantly different between cases and controls, for example, dysphagia and/or odynophagia were significantly more frequent endoscopic indications in cases compared to controls (72.29 % vs 5.56 %, *p* < 0.0001) (Table 2). Overall, the common sites for esophageal masses were the lower and mid portions of the esophagus in 28 (41.79 %) and 21 (31.34 %) respectively. Self-reported HIV infection rates were similar between these groups.

**Table 2:** Endoscopic examination features of cases and controls

Endoscopic features	Control	Case
	*n* = 142, *n* (%)	*n* = 67, *n* (%)
Normal	30 (21.13)	_
Upper esophageal mass	_	5 (7.46)
Mid esophageal mass	1 (0.70)	6 (8.96)
Lower esophageal mass	8 (5.63)	21 (31.34)
Esophageal mass (unspecified location)	2 (1.41)	28 (41.79)
GE junctional mass	4 (2.82)	2 (2.99)
Gastritis	45 (31.69)	1 (0.49)
Others^a^	83 (58.45)	2 (2.99)
Missing	14 (9.86)	2 (2.99)
Rapid urease (CLO) positivity	42 (29.58)	9 (13.43)

Others^a^: gastritis of unspecified location; CLO: Campylobacter like organism

In the univariate logistic regression modeling, we found; increasing age, male gender, alcohol & smoking as factors associated with a diagnosis of ESCC. In the multivariate logistic regression analysis (Table 3), we found male gender (AOR 3.65, 95 % CI (1.67 – 7.98), *p* = 0.001), age group of 41 to 50 years (AOR 12.95, 95 % CI (2.57 – 65.10), *p* = 0.002); 51 – 60 year (AOR 6.50, 95 % CI (1.32 – 31.90), *p* = 0.021); 61 – 70 year (AOR 7.26, 95 % CI (1.45 – 36.41), *p* = 0.016); and age > 70 year (AOR 5.23, 95 % CI (0.99 – 27.69), *p* = 0.052) were independently correlated with esophageal squamous cell carcinoma. Self-reported use of alcohol and smoking were not statistically associated with ESCC (Table 3).

**Table 3:** Univariate and multivariate logistic regression models evaluating risk factors for esophageal squamous cell carcinoma in Southwestern Uganda

Characteristic	Univariate Model OR (95 % CI)	*p*-value	Multivariate Model Adjusted OR (95 % CI)	*p*-value
Female gender	REF		REF	
Male gender	3.33 (1.69 – 6.55)	0.0001	3.65 (1.67 – 7.98)	0.001
Age category				
31 – 40	REF		REF	
41 – 50	9.84 (2.09 – 46.21)	0.004	12.95 (2.57 – 65.10)	0.002
51 – 60	7.25 (1.53 – 34.28)	0.012	6.50 (1.32 – 31.90)	0.021
61 – 70	8.59 (1.80 – 40.92)	0.007	7.26 (1.45 – 36.41)	0.016
> 70	7.25 (1.46 – 36.10)	0.016	5.23 (0.99 – 27.69)	0.052
Substance use^a^				
Never smoke and alcohol	REF		REF	
Smoking	2.93 (1.43 – 5.71)	0.003	1.38 (0.41 – 4.67)	0.600
Alcohol	1.46 (0.76 – 2.82)	0.255	0.91 (0.32 – 2.64)	0.864
Alcohol & smoking	3.49 (1.46 – 8.34)	0.005	1.93 (0.32 – 11.42)	0.471
Rapid urease test positivity	0.23 (0.05 – 0.97)	0.046	-	-
HIV infection	3.68 (0.49 – 27.64)	0.205	-	-

Substance use^a^: self-reported current or former use

To estimate the population attributable fraction of ESCC due to smoking and alcohol, age data was used as continuous (modeling decision taken based on the -2Log likelihood). In the unadjusted models, the population attributable fraction of ESCC due to male gender was 55.36 %, 95 % CI (26.46 – 72.90), female gender was −48.71 %, 95 % CI ( −81.33 – −21.97), and alcohol & smoking was 14.90 %, 95 % CI (2.95 – 25.38). After adjusting for age and gender, the population attributable fraction of ESCC due to a combination of alcohol & smoking was 12.66 %, 95 % CI (−1.29 – 24.61) (Table 4).

**Table 4:** Unajusted and adjusted population attributable fraction of esophageal squamous cell carcinoma due to gender, individual and combined effects of smoking and alcohol in Southwestern Uganda

Characteristic	Crude PAF model	^a^Adjusted PAF model
	% PAF (95 % CI)	% PAF (95 % CI)
Male	55.36 % (26.46 – 72.90)	-
Female	−48.71 % (−81.33 – −21.97)	-
Substance use		
Never smoke and alcohol	REF	REF
Smoking	19.67 % (4.91 – 32.14)	15.62 % (−2.49 – 30.53)
Alcohol	9.04 % (−8.42 – 24.36)	10.17 % (−9.12 – 26.05)
Alcohol & smoking	14.90 % (2.95 – 25.38)	12.66 % (−1.29 – 24.61).

^a^Adjusted for age and gender

## Discussion

This is the first study to report PAFs for ESCC risk factors smoking and alcohol in an ESCC high-risk region in sub-Saharan Africa. Our study describes a low population attributable fraction of esophageal squamous cell carcinoma due to smoking and alcohol use in southwestern Uganda i.e., if smoking and alcohol use were eliminated in the rural population of southwestern Uganda, approximately 13 % of new esophageal squamous cell carcinoma cases could be avoided. This is lower than an estimated two-thirds and three-fourths population attributable fractions of ESCC for smoking and alcohol respectively, from priorstudies in high - risk and low - risk populations [12–15]. However, our results corroborate findings from one of the ESCC highest risk areas of northern China (Linxian County) where it has been suggested that alcohol and tobacco consumption are not the major risk factors for ESCC [21]. We posit that ESCC in this population results from multifactorial interactions of environmental factors, diet, and genetics [22–24] and not individual factors (alcohol and smoking) only.

The PAFs stratified by gender indicated that a higher percentage of ESCC would be prevented by change in gender which confirms that male gender confers a higher risk of ESCC as described by other studies [25, 26] presumably due to the high exposure to smoking and alcohol [26] or hormonal differences [27, 28]. In fact, the PAFs of smoking and alcohol as individual factors indicated that smoking had the highest PAF for ESCC in the unadjusted (19.7 %) as well as in the gender and age adjusted analyses (15.6 %). The PAF estimates for alcohol use were lowest even when compared with the PAF of the combined term of smoking and alcohol. This finding emphasizes that smoking (and less so alcohol use) is more prevalent in endemic regions translating into a high attributable fraction. Therefore, other factors including genetic variations, socioeconomic disparities, dietary factors, and possibly infectious causes, are may contribute to the population attributable fraction differences in this population [29].

Our study highlights ESCC occurring in younger age (<60 years) despite the global trends that esophageal squamous cell cancer is known to occur in the late 6th and 7th decades of life [30] and cases occurring before the age of 40 years are rare [31]. Within Uganda, esophageal cancer has been described in older age (>60 years) in central [32] and northern region [33]. However, our finding is similar to reports from southwestern Kenya, which showed a significant number of esophageal cancer patients presenting at very young ages [31, 34]. This supports our assertion of peculiar risk factors for ESCC that likely afflicts earlier in life such that there is earlier presentation of disease at younger age. Taken together, we posit that maybe there may be unique carcinogenic risk factor(s) for ESCC leading to early onset and/or radid progression.

This data should be interpreted in the context of the study design. As patients studied were referred for endoscopy to a referral center, referral bias likely exists and they may not accurately represent the general population.

As with all non-randomized observational studies, our study could suffer from unmeasured or residual confounding on the associations between younger age (<40 years) and ESCC. However, since our results are similar to findings in high-risk populations, such confounders are more likely to represent actual mediators of the relationship between young age and ESCC, than true confounder *per se*.

## Conclusion

In this study, only 13 % of esophageal squamous cell carcinoma cases would be avoided if alcohol and smoking were discontinued in a rural population of southwestern Uganda, suggesting that important risk factors for esophageal squamous cell carcinoma in this population are not known. Future work should focus on relationships between environmental, infections, diet, genetic, and epigenetic factors as esophageal squamous cell carcinoma risks in this population.
